# 

**Published:** 1888-11-24

**Authors:** 


### CONTENTS

**Medical Monopolies and General Practitioners** - 113

**In and Out Among the Hospitals** - 114

**The Doctor's Pulpit.**—Present Day Problems —The Question of Population - 115

**Sixteen Years of the Hospital Sunday Fund: Its Influence on the Metropolitan Medical Charities.**—By SIR SYDNEY WATERLOW, Bart. - 116

**The Great Meeting at Barts.**—By ONE WHO WAS THERE - 117

**Turning Over New Leaves**—"Massage." - 118

**Voyages in Vacation.**—IX. Impressions of St. Petersburg - 119

**Notes and News** - 120

**Everybody's Page** - 122

**Annotations.**—Competitive Examinations: A Medical Protest—Are Women Deteriorating?—A "Plaintive Cry" - 123

**Within the Wards.**—Alcoholic Paralysis.—A Fright and Its Consequences —Vegetarianism and Cancer - 124

**Socialists and Hospitals.**—By Sir W. MOORE, K.C.I.E. 125

**False Impressions.**—By CAROLINE FOTHERGILL - 126

**A Case to Help** - 127

**Scraps and Gleanings—Amusements and Relaxation** 128

### EXTRA SUPPLEMENT.—THE NURSING MIRROR.

**En Passant.**—The Guild of St. Barnabas.—Menial Work.—The Necessity for Gentleness.—Changes at Charing Cross.—Trained or Not?—The St. Deny's Sisterhood—Nurses and Presents—Another Victoria Home for Nurses - xxix

**Original Articles.**—One Way of Nursing, Part I. - xxx

**Jinny's Twin** - xxx

**Everybody's Opinion.**—Nurses and Presents - xxxi

**"Our Nurse"** - xxxii

**Presentations—Appointments—Lectures** - xxxii

**Vacancies—Notes and Queries** - xxxii

